# Severe Intrahepatic Cholestasis Pregnancy Is Associated With Maternal Endothelial Dysfunction: A Case-Control Study

**DOI:** 10.7759/cureus.32276

**Published:** 2022-12-06

**Authors:** Mehmet Mete Kırlangıç, Erdem Sahin, Mefkure Eraslan Sahin, Yusuf Madendag, Ilknur Col Madendag, Mehmet Ak, Erol Karakas, Sabahattin Muhtaroglu

**Affiliations:** 1 Obstetrics and Gynaecology, Kartal Dr. Lutfi Kirdar City Hospital, Istanbul, TUR; 2 Obstetrics and Gynaecology, Faculty of Medicine, Erciyes University, Kayseri, TUR; 3 Obstetrics and Gynaecology, Kayseri City Hospital, Kayseri, TUR; 4 Biochemistry, Faculty of Medicine, Erciyes University, Kayseri, TUR

**Keywords:** endocan, human endothelial cell-specific molecule 1, endothelium, icp, intrahepatic cholestasis of pregnancy

## Abstract

Objective: The aim of the present study was to evaluate maternal serum endocan levels, which are markers of vascular pathologies and strongly associated with vascular inflammation and endothelial dysfunction, in pregnancies complicated by intrahepatic cholestasis of pregnancy (ICP).

Methods: The study comprised 30 pregnant women with mild ICP, 30 pregnant women with severe ICP, and 30 healthy pregnant women as a control group. The inclusion criteria were women with ICP, which was diagnosed based upon the presence of pruritus associated with elevated total bile acid (TBA) levels (> 10 μm/L), elevated aminotransferases, or both, and the absence of diseases that may produce similar laboratory findings and symptoms. Severe ICP was defined as TBA > 40 μmol/L. After diagnosis for ICP, blood samples were obtained before medication during hospitalization to analyze maternal serum endocan levels.

Results: Gestational age at delivery, delivery induction rates, birth weight, and newborn intensive care unit (NICU) admission rates were significantly higher in the severe ICP group than in the control group and mild ICP group. Gestational age for all groups when blood was sampled was similar. Maternal serum TBAs and aminotransferase levels were significantly higher in the severe ICP group than in the control group and mild ICP group. The mean serum endocan levels were 10.9 ± 2.6 ng/mL in the control group, 12.5 ± 2.8 ng/mL in the mild ICP group, and 24.3 ± 4.8 ng/mL in the severe ICP group (p < 0.001).

Conclusion: Our results indicated that maternal serum endocan levels were increased in the presence of severe ICP and it can be speculated that increased bile acid levels were associated with maternal endothelial dysfunction.

## Introduction

Intrahepatic cholestasis of pregnancy (ICP) is a pregnancy-associated disease that causes unexplained itching on the soles and palms [1]. It is characterized by increased bile acid and liver transaminase levels, usually occurring in the third trimester, and observed in ~0.4%-4% of all pregnancies [1,2]. It has been clearly shown that the severity and frequency of maternal and fetal complications are correlated with increased total bile acid (TBA) levels [3,4]. Higher TBAs are associated with meconium-stained amniotic fluid, premature preterm rupture of membranes, and perinatal death, and it has been reported that every 10-mmol/L increase in TBA increases these complications [4-6].

The endothelium is the smallest endocrine organ comprising a squamous epithelial layer within the lining of the vessel lumen. This organ releases vasodilator and vasoconstrictor substrates to maintain vascular homeostasis [7]. The endothelium blocks specific substances from surrounding tissue to the vessel lumen, provides a non-thrombogenic surface to avoid coagulation, reduces inflammation, and helps control blood pressure through vasoconstriction [7]. Endocan is a vasculoprotective protein and is secreted by the endothelial cells [8]. These cells have an important function in regulating endothelial dysfunction caused by inflammation and protecting the endothelium from inflammatory proliferation [8,9]. Endocan, a marker for vascular pathologies, is strongly related to vascular inflammation, atherosclerosis, and endothelial dysfunction and is reported to be increased in preeclampsia and other gynecologic conditions associated with endothelial dysfunction, such as polycystic ovary syndrome [10-13].

Bile from the liver contains bile acids as the main organic solutes [14]. These acids play various important roles in the intestine and liver, and their retention under specific pathophysiological conditions, such as cholestatic diseases, can cause liver damage by inducing hepatocyte apoptosis or necrosis [14]. It has been reported that increased TBA induces oxidative stress, cell death pathways, and damage to the plasma membranes [14]. This situation has also been observed in autopsies of fetuses who had died from complications related to ICP; it has also been observed that the placentas of patients were adversely affected by acute hypoxia [15]. In the present study, we considered these toxic effects of bile acids and hypothesized based on these effects that elevated TBAs within the maternal vascular area may damage the endothelium. Hence, the aim of the present study was to evaluate maternal serum endocan levels in pregnancies complicated by ICP.

## Materials and methods

This cross-sectional case-controlled study was conducted at Erciyes University Perinatology Clinics, approved by the Ethics Committee of Erciyes University (decision number: 2019-608), and conducted in accordance with the Declaration of Helsinki. All individuals participating in the study provided their informed consent. The study comprised 30 pregnant women with mild ICP, 30 pregnant women with severe ICP, and 30 healthy pregnant women as a control group. The inclusion criteria were women with ICP, which was diagnosed based upon the presence of pruritus associated with elevated total serum bile acid levels (bile acid > 10 μm/L), elevated aminotransferases, or both, and the absence of diseases that may produce similar laboratory findings and symptoms. Severe ICP was defined as bile acids > 40 μmol/L [16]. In the presence of multiple pregnancies, chromosomal abnormalities, thyroid diseases, hypertension, preeclampsia and diabetes mellitus, maternal infection, a history of gallstones or gallbladder disease, drug consumption, hepatitis, or other diseases associated with abnormal liver function, patients were excluded in the study [1]. In addition, pregnant women with COVID-19 were not included in the study. The gestational week was calculated using the first day of the patient’s last menstrual cycle. If not known, ultrasonographic evaluations obtained during the first trimester were used.

After diagnosis for ICP, blood samples were obtained before medication during hospitalization to analyze serum endocan levels. Blood samples from the control group were also taken during the third trimester and admission period. The blood samples were centrifuged at 4,000 rpm for 10 min to separate the serum, which was then frozen at -80°C for subsequent analyses. Human endothelial cell-specific molecule-1 (ESM-1) enzyme-linked immunosorbent assay kit (ELISA; Catalog No. CSB-E16530h) was used to determine maternal serum endocan levels.

In the pilot study leading to this research, maternal serum endocan levels of the mild group (n = 10) and control group (n = 15) were analyzed. The serum endocan levels were 13.1 ± 2.8 in the mild ICP group and 11.3 ± 2.5 in the control group. According to the power analysis, alpha = 0.05, group ratio 1:1, power (1-β) = 0.8, and effect size calculated as 0.678, 28 patients per group were determined as necessary. When a 10% drop rate was added, 30 patients per group were calculated, necessitating 90 patients in total. Statistical analyses were done using Statistical Package for the Social Sciences version 18 (IBM Inc., Armonk, NY, USA) (www.ibm.com/products/spss-statistics). The Kolmogorov-Smirnov test was used for determining the normality of the data. The Levene test was used to evaluate the assumption of variance homogeneity; values are expressed as the mean ± standard deviation, median (min-max), or n (%). p < 0.05 was considered statistically significant. One-way analysis of variance (ANOVA) was performed to compare multiple groups (Tukey’s post hoc test) after evaluating for normal distribution. Comparison of categorical data in paired groups was made by the chi-square test, while the comparison of non-categorical data in paired groups was made by the Mann-Whitney U test.

## Results

Table 1 lists the demographic and delivery characteristics of the participants. There was no intergroup difference among maternal age, BMI, nulliparity, ethnicity, previous cesarean history, and smoking rates (p = 0.910, p = 0.870, p = 0.830, p = 0.960, p = 0.840, and p = 0.450, respectively). Gestational age at delivery, delivery induction rates, birth weight, and newborn intensive care unit (NICU) admission rates were significantly higher in the severe ICP group than in the control group and mild ICP group (p < 0.001, p < 0.001, p < 0.001, and p < 0.001, respectively).

**Table 1:** Comparison of maternal demographics and delivery outcomes between groups. ICP, intrahepatic cholestasis of pregnancy; BMI, body mass index; NICU, newborn intensive care unit. Data are presented as the mean ± SD, median, or n%. ^a^Gestational age at delivery: severe ICP to mild ICP: p < 0.001; severe ICP to control: p < 0.001; and mild ICP to control: p = 0.640. ^b^Delivery induction rates: severe ICP to mild ICP: p < 0.001; severe ICP to control: p < 0.001; and mild ICP to control: p < 0.001. ^c^Birth weight: severe ICP to mild ICP: p < 0.001; severe ICP to control: p < 0.001; and mild ICP to control: p = 0.760.

Demographics	Control Group (n = 30)	Mild ICP Group (n = 30)	Severe Group (n = 30)	p-value
Maternal age (year)	27.8 ± 5.8	28.1 ± 5.2	22.7 ± 5.2	0.910
Nulliparity	10 (33.3)	9 (30)	11 (36.6)	0.870
BMI at blood sampling (kg/m^2^)	28.0 ± 2.3	28.1 ± 2.4	27.2 ± 2.6	0.830
Ethnicity (Caucasian)	28 (93.3)	29 (96.6)	29 (96.6)	0.960
Previous cesarean history	8 (26.6)	9 (30)	8 (26.6)	0.840
Smoking	4 (13.3)	5 (16.6)	5 (16.6)	0.450
Delivery outcomes				
^a^Gestational age at delivery (week)	39 (38-40)	39 (37-39)	36 (36-37)	<0.001
^b^Delivery induction rates	4 (13.3)	12 (40)	19 (63.3)	<0.001
^c^Birth weight (g)	3350 ± 310	3250 ± 380	2950 ± 310	<0.001
Male gender	16 (53.3)	14 (46.6)	17 (56.6)	0.890
Umbilical cord ph	7.29 ± 0.06	7,32 ± 0.04	7.30 ± 0,04	0.246
NICU admission rate	0 (0)	1 (3.3)	5 (16.6)	<0.001

Table 2 lists the biochemical parameters of the participants. The gestational age for all groups when blood was sampled was similar (p = 0.420). Maternal serum TBAs, alanine transaminase (ALT), and aspartate aminotransferase (AST) levels were significantly higher in the severe ICP group than in the control group and mild ICP group (both p < 0.001). We found no significant difference between serum fasting glucose, blood urea nitrogen (BUN), creatinine, sodium, and potassium levels between the two groups. The mean serum endocan levels were 10.9 ± 2.6 ng/mL in the control group, 12.5 ± 2.8 ng/mL in the mild ICP group, and 24.3 ± 4.8 ng/mL in the severe ICP group. The mean serum endocan levels were significantly higher in the severe ICP group than in the control group and mild ICP group (p < 0.001).

**Table 2:** Comparison of biochemical parameters between groups. ICP, intrahepatic cholestasis of pregnancy; ALT, alanine transaminase; AST, aspartate aminotransferase; TBA, total bile acid; BUN, blood urea nitrogen; Na, sodium; K, potassium. Data are presented as the mean ± SD. ^a^Serum endocan levels: severe ICP to mild ICP: p < 0.001; severe ICP to control: p < 0.001; and mild ICP to control: p = 0.880. ^b^Serum ALT: severe ICP to mild ICP: p < 0.001; severe ICP to control: p < 0.001; and mild ICP to control: p < 0.001. ^c^Serum AST: severe ICP to mild ICP: p < 0.001; severe ICP to control: p < 0.001; and mild ICP to control: p < 0.001. ^d^Serum TBA: severe ICP to mild ICP: p < 0.001; severe ICP to control: p < 0.001; and mild ICP to control: p < 0.001.

Biochemical Parameters	Control Group (n = 30)	Mild ICP Group (n = 30)	Severe ICP Group (n = 30)	p-value
Gestational age at blood sampling (week)	34.2 ± 1.7	33.7 ± 1.8	32.7 ± 1.6	0.420
^a^Serum endocan levels (ng/mL)	10.9 ± 2.6	12.5 ± 2.8	24.3 ± 4.8	<0.001
Serum fasting glucose (mg/dL)	80.1 ± 7.3	78.2 ± 8.6	82.4 ± 7.6	0.380
^b^Serum ALT (IU/L)	18.4 ± 3.2	70.8 ± 26.4	102.4 ± 41.1	<0.001
^c^Serum AST (U/L)	16.1 ± 2.1	60.9 ± 24.4	80.3 ± 36.3	<0.001
^d^Serum TBA (μmol/L) (fasting)	4.1 ± 1.2	28.6 ± 8.9	54.2± 10.8	<0.001
Serum BUN (mg/dL.)	7.9 ± 1.8	7.3 ± 2.4	7.9 ± 2.4	0.750
Serum creatinine (mg/dL.)	0.76 ± 0.21	0.69 ± 0.15	0.82 ± 0.21	0.860
Serum Na (mmol/L)	138.6 ± 3.2	137.7 ± 3.1	138.1 ± 3.4	0.780
Serum K (mmol/L)	3.7 ± 0.22	3.9 ± 0.25	3.9 ± 0.20	0.510

## Discussion

As in cholestatic diseases, retention of hydrophobic bile acids can induce hepatocyte apoptosis or necrosis and increases bile acid-induced oxidative stress, cell death pathways, and damage to plasma membranes [14]. In the current study, we hypothesized that the toxic effects of elevated bile acids within the maternal vascular area may damage the endothelium. Our key finding is that maternal serum endocan levels were increased in the severe ICP group compared to the mild ICP group and control group. To our knowledge, this is the first study to report that increased bile acids can cause endothelial damage in patients complicated by ICP.

In the literature, there are few studies and there have been some conflicting results of studies on ICP-related oxidative stress and endothelial dysfunction. Tayyar et al. in a cross-sectional study with 53 women with ICP and 52 healthy controls reported that albumin/albumin ratio modified with ischemia in women with ICP was compared with the control group, and then they reported that they were correlated with the disease severity [2]. In another study, Sanhal et al. [17] evaluated the thiol/disulfide ratio, a marker of oxidative stress, in women with ICP, and observed that the native and total thiol ratios were significantly decreased and disulfide levels were significantly increased. On the contrary, in their study, Krasomski et al. [18] compared 12 patients with cholestasis with 33 healthy pregnant women based on oxidative stress markers and did not find any correlation between the groups; however, the small study group might have been an important limitation of this study. Wang et al. [19] reported that inducible nitric oxide synthase (iNOS) regulates vasodilation of blood vessels, which are factors in ischemic-hypoxic states and that iNOS levels in maternal serum and placenta of women with ICP were significantly lower in controls. In the current study, we found that maternal serum endocan levels were increased in the severe ICP group compared to the mild ICP group and control group. Our results can be explained by the toxic effects of bile acid. It is well documented that higher bile acid has toxic effects on endothelial injury to the lungs and kidneys by releasing reactive oxygen species [14].

The findings of the present study demonstrated clinical importance. First, we must remember the relationship between ICP and preeclampsia. It is well known that ICP increases the incidence of preeclampsia and that severe ICP is a major risk factor for preeclampsia [20]. Raz et al. reported that preeclampsia occurs most often within two to four weeks after ICP diagnosis and is related to ICP severity [20]. In addition, preeclampsia occurs even when the liver enzymes and TBA levels decrease toward their normal ranges. In their systematic review and meta-analysis, Arafa and Dong have shown that ICP is related to the risk of preeclampsia and gestational diabetes mellitus [21]. Antenatal follow-ups are important in terms of late-onset preeclampsia that may develop secondary to endothelial damage in the presence of severe ICP. Second, it has been reported that ursodeoxycholic acid (UDCA) has a proactive effect against bile acid-induced cell injury [14]. UDCA had a critical role in the prevention of oxidative injury with a direct antioxidant effect or an increase in antioxidant defenses [14]. In severe ICP cases, early diagnosis and initiation of treatment using UDCA can protect endothelial damage from higher toxic bile acid levels [14]. Third, cardiology consultation should be requested in the postpartum period in pregnant women who are complicated with severe ICP. This may prevent the resulting endothelial dysfunction from facing adverse maternal vascular outcomes in the future. We are aware of the limitations of our study. We believe that, in our small sample size, cross-sectional design may have limitations to the present study and that additional studies using a larger sample size might provide more information.

## Conclusions

Our results indicated that maternal serum endocan levels were increased in the presence of severe ICP, and it can be speculated that increased bile acid levels were associated with maternal endothelial dysfunction. In the presence of ICP, care should be careful in the peripartum period, and cardiology should be consulted. Further study is needed to clarify this situation.
